# Molecular Signaling Involved in Oxysterol-Induced β_1_-Integrin Over-Expression in Human Macrophages

**DOI:** 10.3390/ijms131114278

**Published:** 2012-11-05

**Authors:** Simona Gargiulo, Paola Gamba, Gabriella Testa, Barbara Sottero, Marco Maina, Tina Guina, Fiorella Biasi, Giuseppe Poli, Gabriella Leonarduzzi

**Affiliations:** Department of Clinical and Biological Sciences, University of Turin, San Luigi Hospital, Regione Gonzole 10, Orbassano 10043, Turin, Italy; E-Mails: simona.gargiulo@unito.it (S.G.); paola.gamba@unito.it (P.G.); gabriella.testa@unito.it (G.T.); barbara.sottero@unito.it (B.S.); marco.maina@unito.it (M.M.); tina.guina@unito.it (T.G.); fiorella.biasi@unito.it (F.B.); gabriella.leonarduzzi@unito.it (G.L.)

**Keywords:** atherosclerosis, oxysterols, β_1_-integrin, cell signaling

## Abstract

The hypercholesterolemia-atherosclerosis association is now established; hypercholesterolemia may induce vascular-cell activation, subsequently increasing expression of adhesion molecules, cytokines, chemokines, growth factors, and other key inflammatory molecules. Among inflammatory molecules expressed by vascular cells, integrins play a critical role in regulating macrophage activation and migration to the site of inflammation, by mediating cell-cell and cell-extracellular matrix interactions. The main lipid oxidation products present in oxidized LDL that may be responsible for inflammatory processes in atherogenesis, are cholesterol oxidation products, known as oxysterols. This study demonstrates the effect of an oxysterol mixture, compatible with that detectable in human hypercholesterolemic plasma, on the expression and synthesis of β_1_-integrin in cells of the macrophage lineage. The molecular signaling whereby oxysterols induce β_1_-integrin up-regulation is also comprehensively investigated. Over-expression of β_1_-integrin depends on activation of classic and novel members of protein kinase C and extracellular signal-regulated kinases 1 and 2, as well as of the up-stream G-protein (Gq and G13), c-Src, and phospholipase C. In addition, the localization of β_1_-integrin in advanced human carotid plaques is highlighted, marking its importance in atherosclerotic plaque progression.

## 1. Introduction

Atherosclerosis is a progressive degenerative process of the arterial blood vessels that is characterized by the accumulation of lipids and fibrous elements in the large and medium arteries. Moreover, it is a multifactorial and multiphasic disease. Today, however, the picture of atherosclerosis is much more complex as it has been considered a chronic inflammatory disease which modulate the initiation and progression of the atherosclerotic lesions [1]; in consequence, any further inflammatory stimulus in the subintimal area automatically becomes a pro-atherogenic stimulus, altering the behavior of the intrinsic cells of the artery wall, and recruiting further inflammatory cells that interact to promote lesion progression and complications. In particular, atherosclerosis is characterized by the activation and migration of monocytic leukocytes into the subendothelial space, and their transformation into lipid-laden macrophage foam cells, both key events in atherogenesis [1]. Monocyte recruitment is a multistep process mediated by adhesion molecules. It begins with rolling, mediated by short-lived bonds between E-selectin on endothelial cells (EC) and sialylated ligands such as P-selectin glycoprotein ligand 1 on monocytes, followed by firm arrest, through interactions between activated β_1_- and β_2_-integrins on monocytes with vascular-cell adhesion molecule-1 (VCAM-1) and intracellular adhesion molecule-1 (ICAM-1) on EC. Firmly arrested monocytes then undergo transmigration through the endothelial monolayer, and subsequent migration through the extracellular matrix (ECM) [2,3].

The association between hypercholesterolemia and atherosclerosis has also been established [4,5]. Hypercholesterolemia contributes to atherogenesis by inducing activation of vascular cells, with the subsequent increased expression of key inflammatory molecules (e.g., adhesion molecules, cytokines, chemokines, and growth factors) and of reactive oxygen species [1,4–9].

Among inflammatory molecules expressed by vascular cells, integrins (heterodimeric αβ transmembrane receptors) play a critical role in regulating monocytes’ adhesion on EC, and their migration through ECM to the site of inflammation, by mediating cell-cell interactions and by connecting the ECM molecules to the cellular cytoskeleton [10–13]. Upon ligand binding, the integrins cluster in distinct microstructures on the plasma membrane; these clusters are known as focal complexes, and may develop into more stable structures, in a condition known as focal adhesion, when the integrins connect with the actin cytoskeleton [13]. Migration of vascular smooth muscle cells (VSMC) from the media into the intima of the artery and their proliferation are also key early events in atherosclerotic lesion development. The interaction between VSMC and ECM molecules is mediated by α_5_β_1_-integrin, a fibronectin receptor which is induced on cell membrane by inflammatory cytokines. This improves VSMC capability of migrating toward ECM [14]. Moreover, it has been demonstrated that α_7_β_1_-integrin apparently promotes the contractile phenotype of VSMC [15].

Binding of several integrins to ECM molecules results in increased expression of various matrix metalloproteinases (MMP), e.g., MMP-1, MMP-2, MMP-3 and MMP-9 [16–18]. Cell migration is intimately linked to interaction with ECM and subsequent degradation of ECM by activated MMP. Some integrins, e.g., α_v_β_3_-integrin, might also regulate vascular permeability in EC during inflammation [19].

Given that numerous oxidative events are associated with the development of atherosclerotic plaques [7], it is now accepted that oxidized low-density lipoproteins (oxLDL), which are taken up by activated macrophages and accumulate in the intima, play a major role in the initiation and promotion of fatty streaks and fibrotic plaques [20,21]. The pro-atherogenic effects of oxLDL are mediated through signaling pathways, which in turn stimulate the expression of genes involved in oxidative stress and the inflammatory response during generation of the atherosclerotic plaque [22,23]. Oxidative stress and inflammation thus go hand-in-hand, because oxidative stress induces the production of inflammatory cytokines, and the cytokines in turn induce free-radical production. Among the several pro-atherogenic effects of oxLDL, the interaction of these lipoproteins with blood monocytes has been found to lead to monocyte differentiation, characterized by β_1_-integrin and β_2_-integrin expression on membranes [9].

The main lipid oxidation products present in oxLDL, which appear to be critically involved in inflammatory and oxidative processes during atherosclerosis development, are cholesterol oxidation products known as oxysterols [24–30]. Confirming oxysterols’ key role in the pathogenesis of atherosclerosis, a significant correlation has been found between total oxysterols recovered from advanced atherosclerotic lesions in the human carotid, and these lesions’ total cholesterol content [27].

These compounds have been directly linked to the induction and propagation of monocytic subendothelial accumulation, and to other inflammatory events associated with the persistent state of vascular inflammation, favoring cellular cross-talk. Moreover, oxysterols stimulate activated macrophages in the atheroma, causing them to produce high levels of cytokines, chemokines, growth factors, adhesion molecules, and other key inflammatory molecules [28,31]. In this connection, an oxysterol mixture compatible with that detectable in human hypercholesterolemic plasma has, in cells of the macrophage lineage, been shown to markedly up-regulate expression and synthesis of the pro-inflammatory mediators transforming growth factor β1 (TGFβ1) [32] and monocyte chemotactic protein-1 (MCP-1) [33], as well as of the scavenger receptor CD36, which is essential in the generation of foam cells [34]. Oxysterols thus appear to be a link between hypercholesterolemia and atherosclerosis through promotion of inflammatory reactions.

On these bases, it was postulated that the same oxysterol mixture might cause strong expression of β_1_-integrin in cells of the macrophage lineage, which is important for monocytic recruitment and differentiation in the subendothelial space during the early stages of atherosclerotic lesion development. The study finds that oxysterols mediate induction of β_1_-integrin in human U937 promonocytic cells. The molecular signaling whereby oxysterols induce β_1_-integrin expression and synthesis is also comprehensively investigated. Moreover, to highlight the involvement of β_1_-integrin in atherosclerotic plaque progression, the localization of this adhesion molecule in advanced human carotid plaques was verified.

## 2. Results and Discussion

### 2.1. Identification of β_1_-Integrin in Human Atherosclerotic Plaques

**Figure 1:**
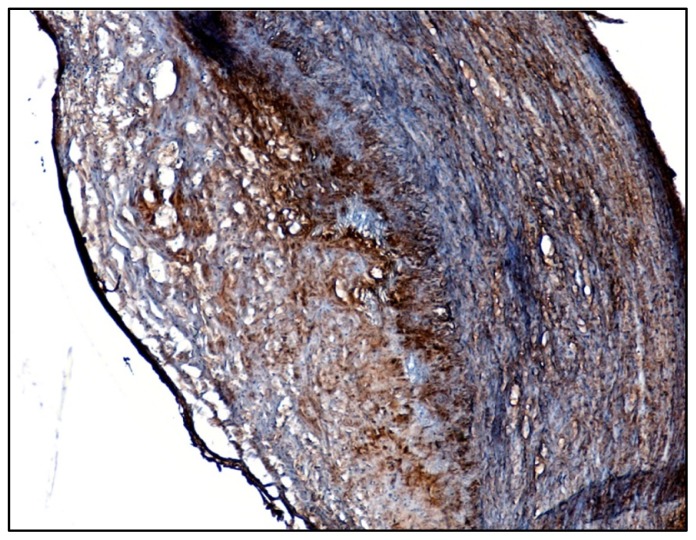
Localization of β_1_-integrin in human atherosclerotic carotid plaques. Artery sections were stained with antibodies against β_1_-integrin; images, observed by light microscopy, were acquired with the “Leica DCF Twain” software package (2.5×). The image is from one representative plaque of five.

In order to investigate the presence of β_1_-integrin in human atherosclerotic lesions, sections of human carotid plaques were used for immunohistochemical analyses. Immunohistochemistry consistently showed large amounts of β_1_-integrin in the core region of the atheromas, within the sub-intimal space as well as in the media of the affected artery (Figure 1). The β_1_-integrin production in the intima should co-localize with macrophages which are the major cells present in the core region while smooth muscle cells should be responsible for its production within the media. These findings were confirmed in five other plaques.

### 2.2. Oxysterols up-Regulate β_1_-Integrin in Human Promonocytic U937 Cells

**Figure 2:**
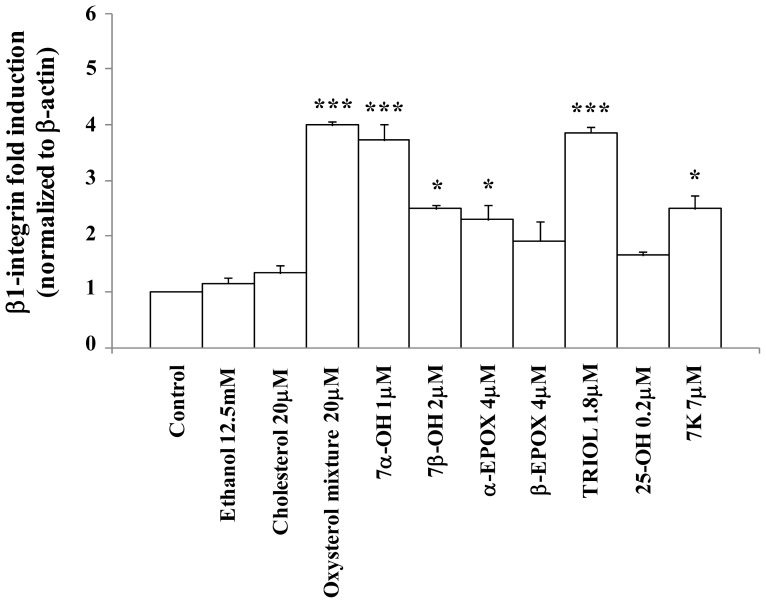
Oxysterols induce β_1_-integrin expression in cells of the macrophage lineage. U937 cells were incubated for 6 h with cholesterol (20 μM), the oxysterol mixture (20 μM), or individual oxysterols at the same concentration present in the 20 μM oxysterol mixture. Untreated cells were used as controls and cells supplemented with 12.5 mM ethanol as solvent controls. Expression of the β_1_-integrin gene was quantified by real-time RT-PCR. Data, normalized to β-actin, are expressed as mean values ± S.D. of three different experiments. ***p < 0.001 and *p < 0.05 *vs.* control; 7α-OH, 7α-hydroxycholesterol; 7β-OH, 7β-hydroxycholesterol; α-EPOX, cholesterol α-epoxide; β-EPOX, cholesterol β-epoxide; TRIOL, cholestan-3β,5α,6β-triol; 25-OH, 25-hydroxycholesterol; 7K, 7-ketocholesterol.

**Figure 3:**
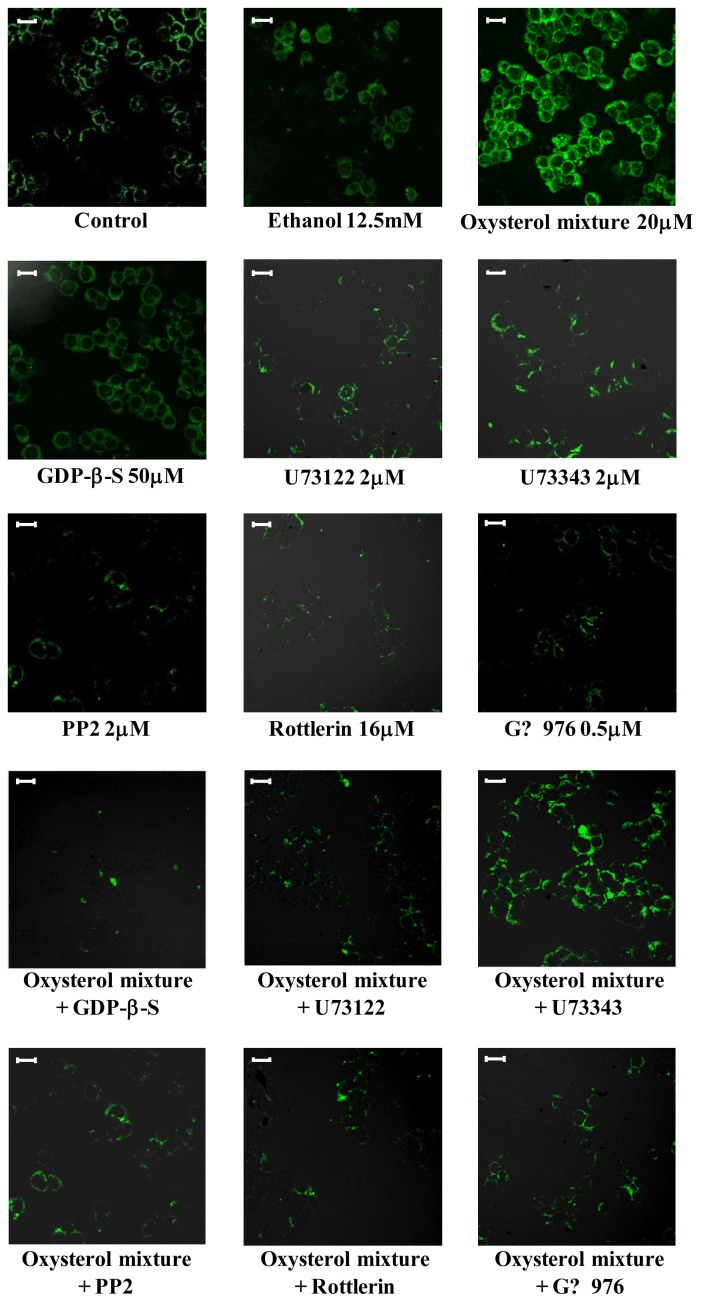
Involvement of the G protein/Src/phospholipase C (PLC)/protein kinase C (PKC) pathway in β_1_-integrin synthesis induced by the oxysterol mixture in cells of the macrophage lineage. U937 cells were incubated for 24 h with the oxysterol mixture (20 μM). Cells were pre-treated with U73122 (2 μM) or with its inactive analogue U73343 (2 μM), or with PP2 (2 μM), or with Rottlerin (16 μM) or with Gö6976 (0.5 μM). Other cells were co-treated with GDP-β-S (50 μM). Untreated cells were used as controls and cells treated with 12.5 mM ethanol or with the various inhibitors were used as internal controls. β_1_-integrin protein levels were detected by confocal laser microscopy using fluorescein isothiocyanate (FITC) fluorochrome (excitation from the 488 nm Ar laser line and emission passing through a long pass 505–550 filter, lens 40×/0.75). Images are from one representative experiment of three. Bars: 20 μM.

To investigate the effect on β_1_-integrin expression of an oxysterol mixture comparable to that found in the plasma of hypercholesterolemic subjects [35–37], human promonocytic U937 cells were incubated with the oxysterol mixture for 6 h, at the non-cytotoxic concentration of 20 μM, or with each individual oxysterol at the concentration present in the 20 μM oxysterol mixture. The effect of the oxysterol mixture on β_1_-integrin mRNA expression was quantified by real time RT-PCR. A significant increase of β_1_-integrin expression occurred (Figure 2). This result is confirmed by the marked induction of β_1_-integrin levels observed by confocal microscopy (Figures 3,5B). Among the different components of the oxysterol mixture, 7α-hydroxycholesterol and cholestan-3β,5α,6β-triol were found to be the main molecules responsible for the observed β_1_-integrin over-expression after 6 h treatment (Figure 2). From these data it appears clear that there is a synergic, rather than an additive, effect among the various oxysterols.

### 2.3. Involvement of the G Protein/Src/PLC/PKC Signaling Pathway in Oxysterol-Mediated Up-Regulation of β_1_-Integrin Levels

Starting from the hypothesis that molecular signaling by oxysterols would likely involve the G protein/Src/phospholipase C (PLC)/protein kinase C (PKC) signaling pathway, as reported elsewhere [38], U937 promonocytic cells were incubated with the oxysterol mixture in the presence or absence of selective inhibitors of that signaling pathway.

**Figure 4:**
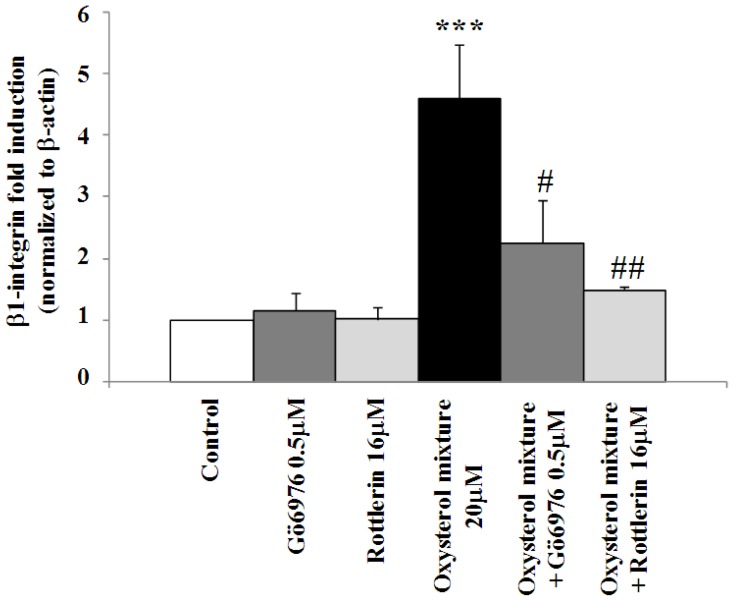
Inhibition of β_1_-integrin over-expression using selective inhibitors of classic and novel PKC isoforms. U937 cells were incubated for 6 h with 20 μM oxysterol mixture; some cells were pre-incubated for 30 min with 0.5 μM Gö6976 or with 16 μM Rottlerin, selective inhibitors of classic and novel PKC isoforms, respectively. Untreated cells were used as controls and cells treated with Gö6976 or with Rottlerin were taken as internal controls. Expression of the β_1_-integrin gene was quantified by real-time RT-PCR. Data, normalized to β-actin, are expressed as mean values ± S.D. of three different experiments. ***p < 0.001 *vs*. control; ## *p* < 0.01 *vs*. the oxysterol mixture; # *p* < 0.05 *vs*. the oxysterol mixture.

The marked induction of β_1_-integrin levels observed when cells were incubated for 24 h with the oxysterol mixture (20 μM) was to a considerable extent prevented by cell co-incubation with GDP-β-S (50 μM), a G protein inhibitor. The same result was obtained through cell pre-treatment with PP2 (2 μM), an inhibitor of tyrosine kinase c-Src, or with U73122 (2 μM), a PLC inhibitor; conversely, *U73343*, an analogue of the latter compound that does not inhibit PLC, did not affect β_1_-integrin levels (Figure 3). The involvement of these signal molecules in β_1_-integrin’s up-regulation by oxysterols presumably entails stimulating members of the PKC family. Using selective chemical inhibitors, *i.e.*, Rottlerin (16 μM) or Gö6976 (0.5 μM) (respectively novel and classic PKCs inhibitors), β_1_-integrin expression (Figure 4) as well as protein levels induced by the oxysterol mixture (20 μM) were also markedly reduced (Figure 3).

The findings support the hypothesis that this signaling pathway is involved in oxysterol mixture-induced β_1_-integrin up-regulation.

### 2.4. Involvement of the ERK1/2 Signaling Pathway in Up-Regulating β_1_-Integrin in U937 Promonocytic Cells

**Figure 5:**
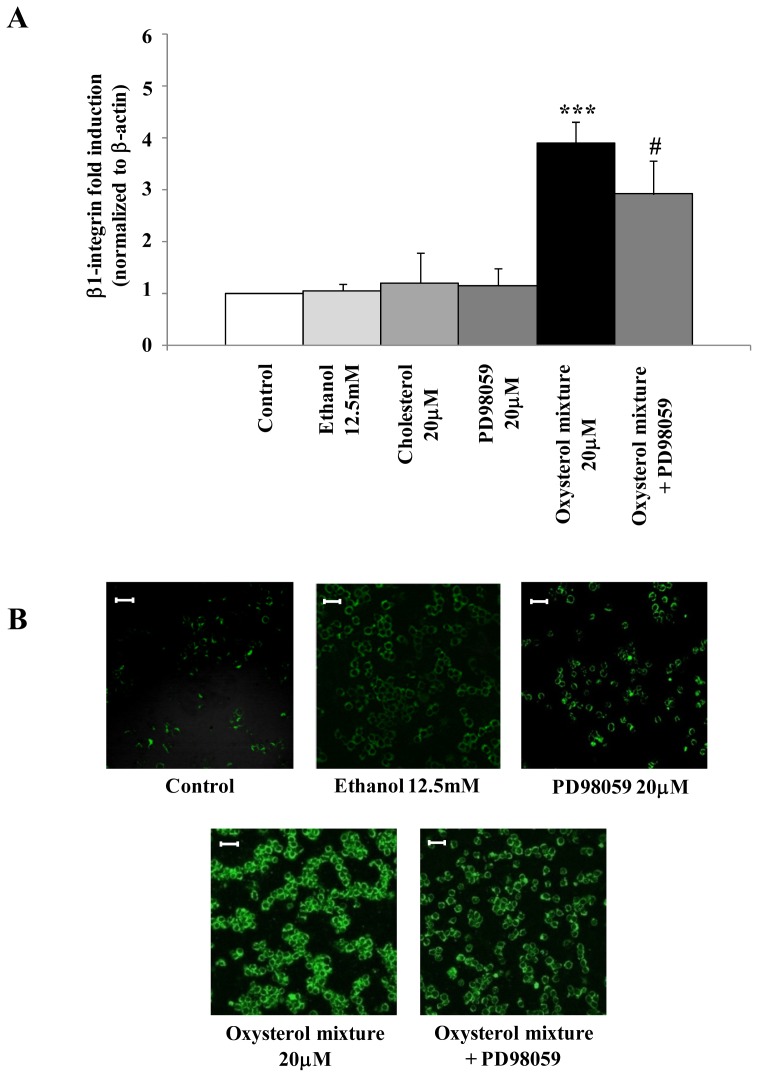
Involvement of the ERK1/2 pathway in β_1_-integrin up-regulation induced by the oxysterol mixture. (**A**) U937 cells were incubated for 6 h with the oxysterol mixture (20 μM), or cholesterol (20 μM). Some cell aliquots were pre-incubated (30 min) with PD98059 (20 μM), a selective inhibitor of MEK1/2. Untreated cells were used as controls and cells treated with 12.5 mM ethanol or with PD98059 were taken as internal controls. *β_1_-integrin* gene expression was quantified by real-time RT-PCR. Data, normalized to β-actin, are expressed as mean values ± S.D. of three different experiments. ***p < 0.001 *vs*. control; # *p* < 0.05 *vs*. the oxysterol mixture; (**B**) Cells were incubated for 24 h with 20 μM oxysterol mixture with or without PD98059 (20 μM) pre-treatment. Untreated cells were used as controls and cells treated with 12.5 mM ethanol or with PD98059 were used as internal controls. β_1_-integrin protein levels were detected by confocal laser microscopy using fluorescein isothiocyanate (FITC) fluorochrome (excitation from the 488 nm Ar laser line and emission passing through a long pass 505–550 filter, lens 20×/0.5). Images are from one representative experiment of three. Bars: 40 μM.

**Figure 6:**
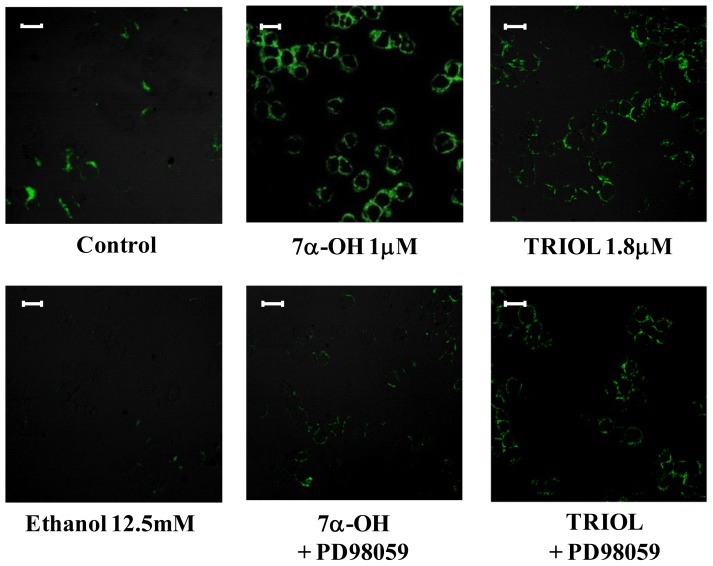
Inhibition of the ERK1/2 pathway decreases β_1_-integrin levels induced by 7α-hydroxycholesterol (7α-OH) and cholestan-3β,5α,6β-triol (TRIOL). Cells were incubated for 24 h with 7α-OH (1 μM) or TRIOL (1.8 μM). Some cell aliquots were pre-incubated for 30 min with PD98059 (20 μM), a selective inhibitor of MEK1/2. Untreated cells and cells treated with 12.5 mM ethanol were used as controls and solvent controls, respectively. β_1_-integrin protein levels were detected by confocal laser microscopy using fluorescein isothiocyanate (FITC) fluorochrome (excitation from the 488 nm Ar laser line and emission passing through a long pass 505–550 filter, 40×/0.75). Images are from one representative experiment. Bars: 20 μM.

To demonstrate that the extracellular signal-regulated kinases 1 and 2 (ERK1/2) signaling pathway play crucial roles in up-regulating β_1_-integrin, U937 cells were pre-incubated with PD98059 (20 μM), a selective mitogen-activated protein kinase/ERK1/2 (MEK1/2) inhibitor. As Figure 5A shows, 6 h cell pre-treatment with PD98059 significantly reduced the β_1_-integrin mRNA expression induced by the oxysterol mixture (20 μM); treatment with cholesterol (20 μM), the unoxidized parent compound, did not affect β_1_-integrin mRNA expression (Figure 5A). Moreover, using the same MEK1/2 inhibitor, β_1_-integrin levels were consistently decreased after 24 h cell treatment with the oxysterol mixture (Figure 5B) and likewise with 7α-hydroxycholesterol (1 μM) or with cholestan-3β,5α,6β-triol (1.8 μM) (Figure 6), at confocal laser microscope observation.

### 2.5. Prevention of β_1_-Integrin Over-Expression in U937 Cells Treated with the Oxysterol Mixture by Cell Transfection with Specific siRNAs for the G Protein/PKC/ERK Pathway

**Figure 7:**
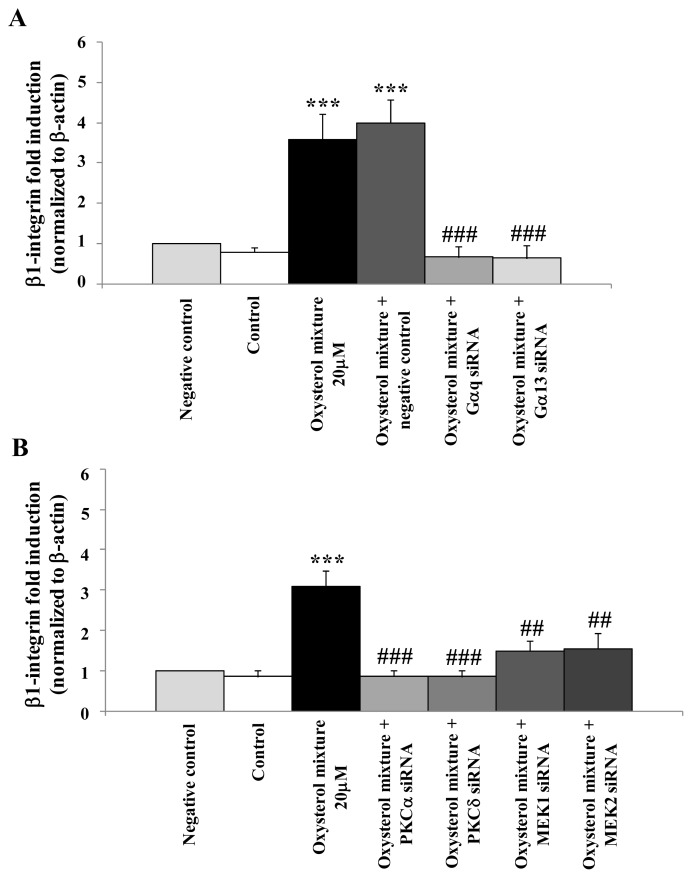
Prevention of oxysterol-induced β_1_-integrin over-expression by G proteins, PKCs or MEK1/MEK2 siRNAs. Verification of Gαq and Gα13 (**A**); PKCα and PKCδ or MEK1/MEK2 (**B**) specific siRNAs (50 nM) on expression of β_1_-integrin in U937 cells treated with the oxysterol mixture (20 μM) for 6 h after reverse transfection (24 h). Untreated cells were used as controls, and cells transfected with unspecific siRNA as negative controls (scramble). Expression of the β_1_-integrin gene was quantified by real time RT-PCR. Data, normalized to β-actin, are expressed as mean values ± S.D. of three experiments. ***p <0.001 *vs.* control; ### *p* < 0.001 and ## *p* < 0.01 *vs.* the oxysterol mixture.

The definitive proof of the crucial involvement of G protein, PKCs and ERK1/2 signal molecules in oxysterol mixture-induced β_1_-integrin expression was provided by specific gene silencing of the G protein (Gαq and Gα13), PKCα, PKCδ, MEK1 or of MEK2. U937 cells were first transfected with the specific siRNAs (50 nM) for 24 h and then incubated for 6 h with the oxysterol mixture. Some cell aliquots were transfected with non-specific RNA sequences (negative controls) or with the oxysterol mixture plus the non-specific RNA sequences, to demonstrate the non-specific effects on gene expression caused by introducing any siRNA. Unlike the oxysterol mixture-treated cells, which after 6 h incubation showed a net increase in β_1_-integrin mRNA, all cells transfected with Gαq and Gα13 siRNAs (Figure 7A) or with PKCα and PKCδ or MEK1 and MEK2 siRNAs (Figure 7B) exhibited a decrease of mRNA levels to values similar to those of controls and negative controls.

### 2.6. Discussion

An important role is here proposed for β_1_-integrin in atherosclerosis development associated with hypercholesterolemia. Significant over-expression of β_1_-integrin was observed in cells of the macrophage lineage after incubation with an oxysterol mixture (20 μM) compatible with that detectable in human hypercholesterolemic plasma. Because of this central role played by oxysterol-induced β_1_-integrin in atherogenesis, it appeared important to investigate the possible signaling pathway involved, in order to provide new molecular insights into the mechanisms of atheroma formation, in the hope of developing new preventative and therapeutic strategies.

As observed in a study regarding monocyte differentiation and scavenger receptor CD36 over-expression induced by oxysterols [8], these oxidized lipids induce up-regulation of β_1_-integrin, a key adhesion molecule for monocyte adhesion and transmigration into the intima, by activating the G-protein/c-Src/PLC/PKC/ERK signaling pathway. The findings obtained by employing selective molecular inhibitors or siRNAs clearly point to the involvement of these signal molecules in oxysterol-mediated signaling which leads to a marked increase of β_1_-integrin expression and levels on human U937 macrophagic cells. Using specific siRNAs, involvement of the Gα_q_ protein and of the Gα_13_ up-stream proteins were demonstrated, as well as that of both classic (PKCα) and novel (PKCδ) members of the PKC family. In agreement with previous studies [33,39,40], only ERK1/2 MAPK appears to be preferentially stimulated by oxysterols and, here, it is shown that ERK1/2 plays a crucial role in oxysterol-β_1_-integrin up-regulation on U937 cells. Finally, to highlight the potential role of β_1_-integrin in atherosclerotic lesion development, the localization of this key adhesion molecule was investigated in advanced human carotid plaques. Large amounts of this adhesion molecule were observed in both the sub-intimal space and in the media of involved arteries, presumably co-localizing with residential macrophages and VSMC, respectively.

On these data, we hypothesize that an oxysterol mixture similar to that found in human hypercholesterolemic plasma might contribute to leukocyte transmigration from the blood circulation to subintimal spaces in the developing atheromasic lesions, by over-expressing β_1_-integrin, a key adhesion molecule mediating cell-cell and cell-ECM interactions.

## 3. Materials and Methods

### 3.1. Immunohistochemical Detection of β_1_-Integrin in Human Carotid Plaques

The histochemical analysis was approved by the local ethical committee and all patients provided their informed consent. Five atherosclerotic carotid plaques were obtained from patients with symptomatic high-grade internal carotid artery stenosis undergoing carotid endarterectomy. The plaques were immediately washed in physiological solution and rapidly frozen using liquid nitrogen. Frozen sections were prepared on a Leica CM 1900 Cryostat for immunohistochemical analyses. Sections of frozen plaques were fixed in 4% formalin; they were incubated with mouse monoclonal antibody to human β_1_-integrin (1:50) (Clone 4B7R, Santa Cruz Biotecnology Inc., Santa Cruz, CA, USA) overnight at 4 °C. For immunohistochemical detection of β_1_-integrin the sections were then incubated with an anti-mouse secondary antibody (1:200) (DakoCytomation, Milano, Italy) for 1 h and then with avidin-biotin complex (1:20) (DakoCytomation, Milano, Italy) for 30 min at room temperature. Finally, to reveal peroxidase activity, sections were incubated in freshly-prepared 0.1% 3,3′-diaminobenzidine (DAB) solution in the dark for 10 min and counterstained with Mayer’s hematoxylin (Sigma-Aldrich, Milano, Italy). The plaque sections were then mounted in DPX (Sigma-Aldrich, Milano, Italy), and observed under a light microscope. Images were acquired using the “Leica DCF Twain” software package.

### 3.2. Cell Culture and Treatments

The human promonocytic cell line U937 was grown in RPMI-1640 (Sigma Aldrich, Milano, Italy) and dispensed as described elsewhere [33]. Cells (1 × 10^6^/mL) were treated with the oxysterol mixture (20 μM) or with unoxidized cholesterol (20 μM) (Steraloids, Newport, RI, USA), both dissolved in ethanol, or with an equivalent volume of ethanol (12.5 mM) used as solvent control. The percentage composition of the oxysterol mixture used was 7α-hydroxycholesterol (5%), 7β-hydroxycholesterol (10%), 5α,6α-epoxycholesterol (20%), 5β,6β-epoxycholesterol (20%), cholestan-3β,5α,6β-triol (9%), 7-ketocholesterol (35%), and 25-hydroxycholesterol (1%). This percentage composition was determined on the quantification of oxysterols in the plasma of hypercholesterolemic patients [35–37]. In certain experiments, the oxysterols were tested individually and added to cell cultures at the concentration present in the 20 μM oxysterol mixture. For chemical inhibitor studies, cells were pre-treated with PP2 (2 μM), a tyrosine kinase c-Src inhibitor, or with U73122 (2 μM), a PLC inhibitor, or with PD98059 (20 μM), a selective MEK1/2 inhibitor (Calbiochem-Merck, Darmstadt, Germany); other cells were co-treated either with GDP-β-S (50 μM), a protein G inhibitor, or with Rottlerin (16 μM) or with Gö6976 (0.5 μM), respectively novel or classic PKCs inhibitors (Calbiochem-Merck, Darmstadt, Germany). Incubation times for all experiments are reported in the figure legends.

### 3.3. RNA Extraction

Total RNA was extracted from treated cells using TRIzol Reagent (Applied Biosystems, Monza, Italy) following the manufacturer’s instructions. RNA was dissolved in RNase-free water fortified with RNase inhibitors (RNase SUPERase-In, Ambion, Austin, TX, USA). The amount and purity (A260/A280 ratio) of the extracted RNA were assessed spectrophotometrically.

### 3.4. cDNA Preparation and Real-Time RT-PCR

cDNA was synthesized by reverse transcription from 2 μg RNA with a commercial kit and random primers (High-Capacity cDNA Reverse Transcription Kit, Applied Biosystems, Monza, Italy) following the manufacturer’s instructions. Singleplex real-time RT-PCR was performed on 25 ng of cDNA using TaqMan Gene Expression Assay kits prepared for human β_1_-integrin and β-actin, TaqMan Fast Universal PCR Master Mix, and 7500 Fast Real-Time PCR System (Applied Biosystems, Monza, Italy). Negative controls did not include RNA. The oligonucleotide sequences are not revealed by the manufacturer, due to proprietary interests. The cycling parameters were as follows: 20 s at 95 °C for AmpErase UNG activation, 3 s at 95 °C for AmpliTaq Gold DNA polymerase activation, 40 cycles of 3 s at 95 °C (melting), and 30 s at 60 °C (annealing/extension). The fractional cycle number at which fluorescence passes the threshold in the amplification plot of fluorescence signal *versus* cycle number was determined for each gene considered. The results were then normalized to the expression of β-actin, as housekeeping gene. Relative quantification of target gene expression was achieved with a mathematical method [41].

### 3.5. siRNA Transfection

Small interfering RNA (siRNA) was used for transient gene knockdown studies. Transfection of Gαq, Gα13, PKCα, PKCδ, MEK1 and MEK2 specific and of negative-control siRNAs was carried out following the manufacturer's instructions (Ambion, Austin, TX, USA). The siRNAs used were GNAq siRNA, GNA13 siRNA, PRCKA siRNA, PRCKD siRNA, MAP2K1 siRNA, MAP2K2 siRNA, and negative control (scramble) siRNA (Ambion, Austin, TX, USA). Briefly, 50 nM of siRNAs were mixed with 25 μL of transfection reagent solution (NeoFX, Ambion, Austin, TX, USA) and left at room temperature for 10 min. After 24 h of reverse transfection, the cells (4 × 10^4^/mL) were centrifuged and incubated with oxysterol mixture 20 μM for 6 h in replaced medium with 2% fetal bovine serum. For gene expression analysis, total RNA was isolated from the cells and used for quantitative RT-PCR as described above. The knock-down efficiency, validated by quantitative RT-PCR, was approximately 60%–70%.

### 3.6. Analysis of β_1_-Integrin by Confocal Laser Microscopy

After treatments, cells were transferred onto glass slides (8 × 10^4^ cells/slide) by cytocentrifugation. Specimens were fixed in 95% ethanol and then incubated in a 100 mM sodium cyanoborohydride reducing agent. After blocking non-specific binding with bovine serum albumin (3%) plus normal goat serum (5%) solution, the slides were incubated in the presence of mouse monoclonal antibody to human β_1_-integrin (1:2000) (Clone 4B7R, Santa Cruz Biotecnology Inc., Santa Cruz, CA, USA) and then with purified goat anti-mouse fluorescein isothiocyanate (FITC) fluorochrome-conjugated secondary antibodies (1:300) (Alexa Fluor 488, Molecular Probes-Invitrogen Srl). Slides mounted with MOWIOL 4-88 (Calbiochem-Merck, Darmstadt, Germany) were observed through the LSM 510 confocal laser microscope (Carl Zeiss SpA, Arese, Milano, Italy).

### 3.7. Statistical Analysis

All values are expressed as means ± standard deviation (S.D.). Statistical analysis of the data was assessed by using one-way ANOVA with Bonferroni’s post test for multiple comparisons and the t test with Welch correction for comparison between two groups. Differences with *p* < 0.05 were considered statistically significant. Statistical calculations were carried out with GraphPad InStat3 software (GraphPad Software Inc., San Diego, CA, USA).

## 4. Conclusion

The present study demonstrated that an oxysterol mixture compatible with that detectable in human hypercholesterolemic plasma induces β_1_-integrin levels in cells of the macrophage lineage, which is important for monocytic recruitment in the intima and their differentiation during the atherosclerotic lesion development. The molecular signaling whereby oxysterols induce β_1_-integrin expression and synthesis has also been clarified, although further studies will be needed to better investigate the mechanisms of action of oxysterols in order to provide new molecular insights into the mechanisms of atheroma formation, in the hope of developing new preventative and therapeutic strategies.
